# The *TCF7L2* rs7903146 polymorphism, dietary intakes and type 2 diabetes risk in an Algerian population

**DOI:** 10.1186/s12863-014-0134-3

**Published:** 2014-12-10

**Authors:** Hadjira Ouhaibi-Djellouli, Sounnia Mediene-Benchekor, Sarah Aïcha Lardjam-Hetraf, Imane Hamani-Medjaoui, Djabaria Naima Meroufel, Houssam Boulenouar, Xavier Hermant, Nadhira Saidi-Mehtar, Philippe Amouyel, Leila Houti, Louisa Goumidi, Aline Meirhaeghe

**Affiliations:** Laboratoire de Génétique Moléculaire et Cellulaire, Université des Sciences et de la Technologie d’Oran Mohamed Boudiaf, Oran, Algeria; Département de Biotechnologie, Faculté des Sciences de la Nature et de la Vie, Université d’Oran, Oran, Algeria; Caisse Nationale des Assurances Sociales des Travailleurs Salariés, Clinique Spécialisée en Orthopédie et Rééducation des Victimes des Accidents de Travail, Oran, Algeria; INSERM, U744; Institut Pasteur de Lille, Université de Lille, 1 rue du Pr. Calmette, BP 245, F-59019 Lille, France; Faculté de Médecine, Université Djillali Liabes de Sidi Bel Abbès, Sidi Bel Abbès, Algeria; Laboratoire des Systèmes d’Information en Santé, Université d’Oran, Oran, Algeria

**Keywords:** Diabetes, Gene-diet interaction, Polymorphism, TCF7L2, ISOR study

## Abstract

**Background:**

The transcription factor 7-like 2 (TCF7L2) gene is the most significant genetic risk factor for type 2 diabetes (T2D). Association analyses were performed on participants (n = 751, aged between 30 and 64) in the ISOR population-based study in the city of Oran. Dietary intakes were estimated using a weekly food frequency questionnaire.

**Results:**

The T allele of the rs7903146 single nucleotide polymorphism (SNP) was associated with lower body weight (*p* = 0.02), lower BMI (*p* = 0.009), lower waist circumference (*p* = 0.01) and a lower waist-to-hip ratio (*p* = 0.02). The T allele was associated with a significantly higher risk of T2D (odds ratio (OR) (95% confidence interval) = 1.55 (1.09–2.20), *p* = 0.01) and this association was independent of BMI. When considering the T2D risk, there were nominal interactions between the rs7903146 SNP and dessert (*p* = 0.05) and milk intakes (*p* = 0.01). The T2D risk was greater in T allele carriers with high dessert and milk intakes (OR = 2.61 (1.51-4.52), *p* = 0.0006, and 2.46 (1.47-4.12), *p* = 0.0006, respectively). In subjects with a high dessert intake, the T allele was also associated with higher fasting plasma glucose concentrations (4.89 ± 0.46 mmol/L in TT subjects, 4.72 ± 0.48 mmol/L in CT subjects and 4.78 ± 0.51 mmol/L in CC subjects; *p* = 0.03).

**Conclusions:**

The T allele of the rs7903146 SNP is associated with a significantly higher risk of T2D in an Algerian population. This association was further strengthened by a high dessert intake, suggesting that gene-diet interactions increase the T2D risk.

## Background

Type 2 diabetes mellitus (T2D) is characterized by hyperglycemia as a result of impaired insulin secretion, insulin resistance in peripheral tissues and/or increased glucose output by the liver [1]. As observed worldwide, the prevalence of diabetes is increasing in Algeria. Indeed, the prevalence has almost doubled over the last 20 years (from 6.9% in 1990 [2] to 13% in 2007 [3]). The increase might be due to the rapid changes in lifestyle recently observed in this Northern African country.

Setting aside the rare monogenic forms of the disease [1], T2D arises from interactions between the patient’s genetic background and his/her environment. An association between rs7903146 of the transcription factor 7-like 2 (*TCF7L2*) gene and the T2D risk was identified for the first time in a study of Icelandic, American and Danish subjects [4], and has since been consistently replicated in various European and non-European populations (including Indian and Japanese populations) [5-9]. In Arab populations, strong associations have been observed in Tunisian [10,11], Moroccan, Palestinian [12], Iranian [13] and Lebanese [14] studies.

To the best of our knowledge, there are no published data on the impact of the *TCF7L2* rs7903146 SNP in Algerian populations. Hence, we decided to evaluate the associations between this SNP, the T2D risk and quantitative metabolic phenotypes in a sample of the Algerian population (the ISOR (*Insulino-résistance à Oran*) study). We also looked at whether or not dietary intakes could modulate these putative associations.

## Methods

### Subjects

The ISOR population-based, cross-sectional study was performed between 2007 and 2009. It investigated a representative sample of 787 subjects (378 men and 409 women, aged between 30 and 64) recruited from within the city of Oran in western Algeria. Subjects were selected at random from social security registers. A questionnaire on lifestyle (physical activity, tobacco use and alcohol intake), personal and family medical histories, current medication and socio-economic and educational levels was completed during a face-to-face interview. The study has been described in detail elsewhere [15].

Food intake was assessed with a weekly food frequency questionnaire: consumption of butter, peanuts, desserts (i.e. sweet dishes, including pastries, custards, pudding, sorbets, etc.), fruits, dried fruits, olive oil, other oils, milk, vegetables, dried vegetables, eggs, bread, potatoes, pasta, fish, chicken and meat products during the week before administration of the questionnaire was noted and then expressed as the frequency of intake (per day or per week). After calculating the median value for the intake of each food type, we defined two groups: non-/low consumers (i.e. those whose intake was below the median value) and moderate/high consumers (i.e. those whose intake was greater than or equal to the median value).

The homeostasis model assessment insulin resistance (HOMA-IR) index was calculated as (fasting plasma glucose (mmol/L) × fasting plasma insulin (mIU/L)/22.5) [16]. The homeostasis model assessment of beta-cell function (HOMA-B) was calculated as (20 × fasting plasma insulin (mIU/L)/fasting plasma glucose (mmol/L)-3.5) [17]. Of the 751 genotyped subjects in the ISOR study, 76 had T2D (defined according to the American Diabetes Association (ADA) criteria, i.e. fasting plasma glucose ≥ 7.0 mmol/L and/or treatment for diabetes, including diets and/or oral antidiabetic drugs and/or insulin for the achievement of glycemic control [18,19]) and 644 did not. Seventeen of the 76 diabetics had not been diagnosed prior to the ISOR study.

### Ethics

The study’s objectives and procedures were approved by the independent ethics committee (*Agence Thématique de Recherche en Sciences de la Santé*, Oran, Algeria). The datasets were anonymized and the subjects’ names, initials or hospital identification numbers were not used. All subjects gave their written, informed consent to participation.

### Genotyping

Genomic DNA was extracted from white blood cells by using a Stratagene® kit (Agilent Technologies, Les Ulis, France), according to the manufacturer’s protocol. The *TCF7L2* rs7903146 SNP was genotyped using KASPar technology (a competitive allele-specific polymerase chain reaction incorporating a fluorescent resonance energy transfer quencher cassette (KBioscience, Hoddesdon, UK). The KASPar assay was designed using Primer Picker software (KBioscience). Genotyping assays was carried out with a Hydrocycler (Applied Biosystems, Foster City, CA) in a final volume of 2 μl containing 4× Reaction Mix (KBioscience), 120 nM of each allele-specific primer, 300 nM of common primer, 1.5 μl of Master Mix (KBioscience) and 5 ng of genomic DNA. The following thermal cycling profile was used: 15 min at 94°C; 20 cycles of 10 s at 94°C, 5 s at 57°C and 10 s at 72°C; and 18 cycles of 10 s at 94°C, 20 s at 57°C and 40 s at 72°C. The specific probe sequence was [AGCACTTTTTAGATA[C/T]TATATAATTTAATTG]. The genotyping success rate was 96%. Appropriate negative control samples were used, including two non-DNA controls per 96-well plate. Around 9% of the participants in the ISOR study were genotyped twice, with a concordance rate of 100%.

### Statistical analyses

Statistical analyses were performed with SAS software (version 9.1, SAS Institute Inc., Cary NC). Hardy-Weinberg equilibrium was tested using a chi-squared test with one degree of freedom. Allele frequencies and genotype distributions in T2D and control groups were compared using Pearson’s chi-squared test. Intergroup comparisons of means were performed with a general linear model (GLM). In order to obtain normal data distributions, fasting glucose, insulin and triglyceride concentrations and HOMA-IR and HOMA-B indices were log-transformed. Interaction with dietary intakes was tested by adding an SNP × food intake interaction term to the GLM model. Odds ratios (ORs) were calculated in multivariate logistic regression analyses. The adjustment variables were age, gender, smoking status and level of physical activity (plus BMI for biochemical and clinical variables).

Bonferroni correction for multiple testing was used to adjust gene-diet interaction analyses and the threshold for statistical significance was set to p ≤ 0.0029 (i.e. 0.05 divided by the seventeen food items). For other tests, the threshold for statistical significance was set to p ≤ 0.05.

## Results

### Characteristics of the study subjects

The clinical characteristics of the study subjects’ are summarized in Table 1. The prevalence of T2D in the ISOR study was 10.5%. The T2D subjects were older than non-T2D subjects (*p* = 9.4 × 10^−15^). The T2D and non-T2D subjects had similar mean total cholesterol, high-density lipoprotein (HDL)-cholesterol and low-density lipoprotein (LDL)-cholesterol values. However, T2D subjects had significantly higher anthropometric parameters, fasting glucose (*p* = 5.71 × 10^−176^), fasting insulin (*p* = 0.002), HOMA-IR (*p* = 4.5 × 10^−25^), HOMA-B (*p* = 6.8 × 10^−37^), triglyceride (*p* = 4.4 × 10^−11^) concentrations, systolic blood pressure (SBP) values (*p* = 6.3 × 10^−8^) and diastolic blood pressure (DBP) values (*p* = 4.5 × 10^−5^) than non-T2D subjects.

**Table 1:** **Clinical characteristics of the genotyped subjects in the ISOR study**

**Parameter**	**Non-T2D subjects**	**T2D subjects**	***p***
	**(n = 644)**	**(n = 76)**	
Age (years)	42.8 ± 9.6	52.0 ± 9.5	9.4 × 10^−15^
Weight (kg)	70.6 ± 14.6	75.6 ± 13.9	0.005
BMI (kg/m^2^)	25.8 ± 5.1	27.9 ± 5.2	0.001
Waist (cm)	86.4 ± 12.2	95.6 ± 10.9	5.8 × 10^−10^
Hip (cm)	101.7 ± 9.7	102.5 ± 11.0	0.53
Waist-to-hip ratio	0.85 ± 0.08	0.94 ± 0.11	9.0 × 10^−17^
Fasting glucose (mmol/L)	4.78 ± 0.49	9.58 ± 3.02	5.71 × 10^−176^
Fasting insulin (μIU/mL)	7.94 ± 5.67	11.34 ± 10.70	0.002
HOMA-IR	1.7 ± 1.2	4.7 ± 5.2	4.5 × 10^−25^
HOMA-B	144.7 ± 130.3	51.8 ± 55.9	6.8 × 10^−37^
Triglycerides (mmol/L)	1.11 ± 0.47	1.58 ± 0.70	4.4 × 10^−11^
Total cholesterol (mmol/L)	4.44 ± 0.90	4.54 ± 1.03	0.33
HDL-cholesterol (mmol/L)	1.26 ± 0.31	1.21 ± 0.30	0.17
LDL-cholesterol (mmol/L)	2.68 ± 0.85	2.67 ± 1.03	0.92
SBP (mmHg)	122.1 ± 17.2	133.6 ± 22.2	6.3 × 10^−8^
DBP (mmHg)	76.2 ± 9.9	81.1 ± 9.2	4.5 × 10^−5^

Data are expressed as the mean ± standard deviation.

### Associations with T2D risk and quantitative metabolic traits

The frequencies of the CC, CT, and TT genotypes of the *TCF7L2* rs7903146 SNP in the ISOR study (n = 751) were 0.34, 0.46 and 0.20, respectively (Table 2). This genotype distribution conformed to Hardy-Weinberg equilibrium (*p* = 0.07). The frequency of the minor T allele was 0.43. The T2D subjects and non-T2D subjects differed significantly in terms of the *TCF7L2* rs7903146 genotype distribution (*p* = 0.04) and allele distribution (*p* = 0.02) (Table 2). The T allele was more frequent in T2D subjects than in non-T2D subjects and was significantly associated with a higher risk of T2D (OR (95% confidence interval (CI)) = 1.55 (1.09–2.20); *p* = 0.01 in an additive model). Further adjustment for BMI did not substantially alter this association (OR = 1.62 (1.13–2.31), *p* = 0.008).

**Table 2:** **Association between the** ***TCF7L2*** **rs7903146 SNP and the T2D risk in the ISOR study**

	**ISOR**	**Non-T2D subjects**	**T2D subjects**		**Additive model (T vs C allele)**		**Dominant model (CT + TT vs CC)**		**Recessive model (TT vs CC + CT)**	
	**n (frequency)**	**n (frequency)**	**n (frequency)**	***p***	**OR (95% CI)**	***p***	**OR (95% CI)**	***p***	**OR (95% CI)**	***p***
n	720	644	76							
CC	244 (0.39)	228 (0.35)	16 (0.21)							
CT	328 (0.45)	287 (0.45)	41 (0.54)	0.04	1.55 (1.09-2.20)*	0.01*	2.26 (1.24-4.13)*	0.008*	1.46 (0.80-2.64)*	0.22*
TT	148 (0.20)	129 (0.20)	19 (0.25)		1.62 (1.13-2.31)^†^	0.008^†^	2.38 (1.30-4.36)^†^	0.005^†^	1.54 (0.85-2.82)^†^	0.16^†^
C allele	818 (0.57)	743 (0.58)	73 (0.48)	0.02						
T allele	624 (0.43)	545 (0.42)	79 (52)							

The odds ratios (OR) (95% confidence interval (CI)) and the *p* values were obtained from logistic regression analyses using additive, dominant or recessive models, adjusted for *age, gender, physical activity and smoking status or ^†^age, gender, physical activity, smoking status and BMI.

We also searched for associations between the rs7903146 SNP and anthropometric variables (BMI, body weight, waist circumference and waist-to-hip ratio), biochemical variables (fasting glucose, fasting insulin and lipid concentrations, HOMA-IR and HOMA-B) and clinical variables (SBP and DBP) in non-T2D subjects from the ISOR study (n = 644) (Table 3). The T allele of the rs7903146 SNP was significantly associated with lower body weight (*p* = 0.02), BMI (*p* = 0.009), waist (*p* = 0.01) and waist-to-hip ratio (*p* = 0.02). HOMA-B and DBP values were respectively lower and higher in T allele carriers than in C allele carriers, although these differences were not statistically significant (*p* = 0.08 for both).

**Table 3:** **Association between the** ***TCF7L2*** **rs7903146 SNP and anthropometric, biochemical and clinical parameters in non-T2D subjects in the ISOR study**

**Parameter**	**CC**	**CT**	**TT**	***p***
	**(n = 228)**	**(n = 287)**	**(n = 129)**	
Weight (kg)	72.1 ± 14.0	70.7 ± 15.2	67.7 ± 14.1	**0.02**
BMI (kg/m^2^)	26.4 ± 5.0	25.7 ± 5.1	24.9 ± 5.1	**0.009**
Waist (cm)	88.1 ± 12.2	86.1 ± 12.2	84.3 ± 11.9	**0.01**
Hip (cm)	102.5 ± 9.7	101.6 ± 9.6	100.6 ± 9.9	0.06
Waist-to-hip ratio	0.86 ± 0.09	0.85 ± 0.08	0.84 ± 0.08	**0.02**
Fasting glucose (mmol/L)	4.80 ± 0.50	4.75 ± 0.50	4.82 ± 0.49	0.34
Fasting insulin (μIU/mL)	8.58 ± 6.67	7.91 ± 5.40	6.88 ± 4.00	0.25
HOMA-IR	1.8 ± 1.4	1.7 ± 1.2	1.5 ± 0.9	0.36
HOMA-B	160.9 ± 172.5	144.7 ± 112.1	114.4 ± 72.9	0.08
Triglycerides (mmol/L)	1.14 ± 0.49	1.14 ± 0.45	1.02 ± 0.47	0.24
Total cholesterol (mmol/L)	4.43 ± 0.93	4.46 ± 0.89	4.38 ± 0.89	0.95
HDL-cholesterol (mmol/L)	1.25 ± 0.32	1.24 ± 0.29	1.30 ± 0.34	0.53
LDL-cholesterol (mmol/L)	2.67 ± 0.86	2.71 ± 0.85	2.64 ± 0.84	0.84
SBP (mmHg)	122.0 ± 16.2	122.5 ± 18.1	121.5 ± 16.9	0.23
DBP (mmHg)	75.9 ± 9.1	76.5 ± 10.8	76.2 ± 9.5	0.08

Data are presented as the mean ± standard deviation.

*p* values were adjusted for age, gender, smoking status and physical activity (plus BMI for biochemical and clinical variables). Statistically significant *p* values are indicated in bold type.

### Gene-diet interactions

**Figure 1:**
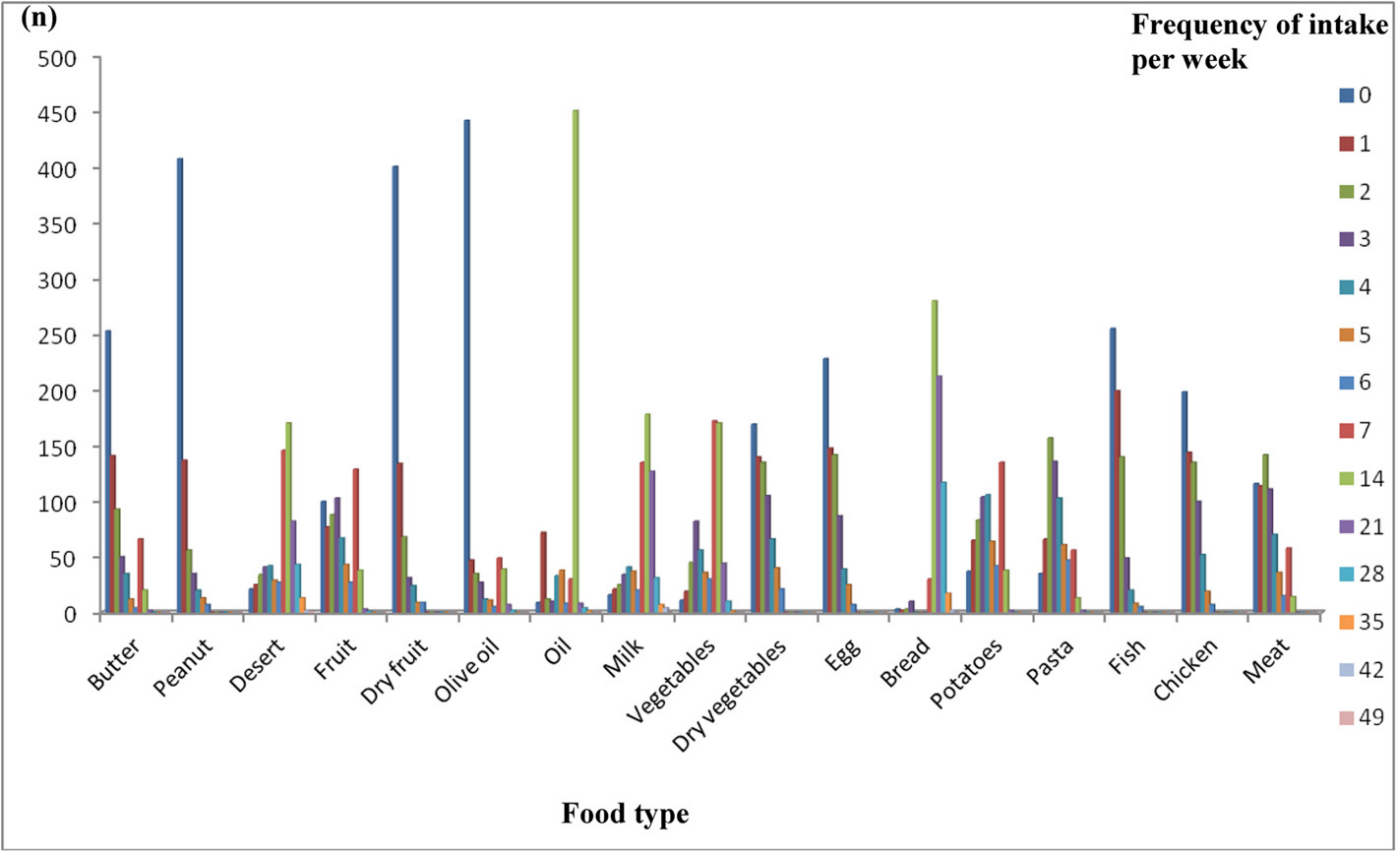
**Weekly intakes of each food type in the ISOR study.** Dietary intakes were estimated with a weekly food frequency questionnaire. The consumption of various food types during the week preceding the administration of the questionnaire was noted and then expressed as the frequency of consumption (per day or per week). For each food type, the number of subjects corresponding to each weekly frequency is shown.

Next, as an exploratory approach, we looked at whether the subjects’ dietary intakes might influence the associations described above. In the ISOR study, we observed high intakes of desserts (consumed at least once a day by 67.6% of the subjects), milk (consumed at least twice a day by 51.3% of the subjects), bread (consumed at least twice a day by 92.9% of the subjects) and vegetable oil (consumed at least twice a day by 66.7% of the subjects) (Figure 1) and very low intakes of olive oil and fish.

For each food type, the subjects were dichotomized into non-/low consumers or moderate/high consumers as a function of the median dietary intake value. When considering the T2D risk, we detected nominal interactions between the *TCF7L2* rs7903146 SNP on one hand and dessert and milk intakes (*p* = 0.05 and *p* = 0.01, respectively) on the other (Table 4). In subjects with a high dessert intake (≥ once a day) or milk intake (≥ twice a day), the rs7903146 SNP was strongly associated with T2D (OR = 2.61 (1.51-4.52), *p* = 0.0006 for desserts; OR = 2.46 (1.47-4.12), *p* = 0.0006 for milk). Conversely, an elevated risk was not observed in non-/low consumers of desserts and milk. Despite the absence of significant interactions with other types of food (relative to the population as a whole; OR = 1.62 for T allele carriers), the T2D risk was even higher in non-/low consumers of peanuts, fruits, dried vegetables, pasta and chicken and in moderate/high consumers of butter, olive oil, other oils and bread (ORs > 2) (Table 4).

**Table 4:** **The association between the rs7903146 SNP and the T2D risk, as a function of food type intakes**

		**Non-/low consumers**				**Consumers or high consumers**			
**Food item**	**Interaction** ***p***	**Non-T2D (n)**	**T2D (n)**	**OR (95% CI)**	***p***	**Non-T2D (n)**	**T2D (n)**	**OR (95% CI)**	***p***
Butter	0.10	237	37	1.14 (0.66-1.98)	0.63	404	39	1.98 (1.23-3.20)	0.005
Peanuts	0.62	386	47	1.76 (1.10-2.82)	0.02	255	29	1.41 (0.81-2.43)	0.22
Desserts	0.05	207	42	1.13 (0.68-1.86)	0.63	434	34	2.61 (1.51-4.52)	**0.0006**
Fruits	0.31	254	25	2.04 (1.11-3.76)	0.02	387	51	1.40 (0.90-2.16)	0.01
Dried fruits	0.54	379	42	1.41 (0.87-2.29)	0.16	262	34	1.70 (0.98-2.95)	0.06
Vegetables	0.12	269	37	1.16 (0.69-1.94)	0.57	372	39	2.11 (1.28-3.50)	0.0036
Dried vegetables	0.61	292	42	1.72 (1.07-2.77)	0.03	348	34	1.50 (0.88-2.55)	0.14
Olive oil	0.50	417	49	1.41 (0.92-2.16)	0.12	224	27	1.98 (1.05-3.75)	0.03
Other oils	0.10	202	29	1.03 (0.56-1.89)	0.93	439	47	1.98 (1.27-3.07)	0.003
Milk	0.01	311	37	1.05 (0.62-1.77)	0.86	330	39	2.46 (1.47-4.12)	**0.0006**
Eggs	0.93	214	28	1.59 (0.91-2.78)	0.11	427	48	1.64 (1.04-2.60)	0.03
Bread	0.50	316	33	1.40 (0.82-2.40)	0.22	325	43	1.92 (1.18-3.12)	0.009
Potatoes	0.69	278	36	1.67 (0.99-2.81)	0.06	363	40	1.47 (0.91-2.38)	0.11
Pasta	0.36	243	31	1.88 (1.06-3.33)	0.03	398	45	1.44 (0.91-2.27)	0.12
Fish	0.76	244	25	1.33 (0.71-2.48)	0.37	397	51	1.68 (1.09-2.58)	0.02
Chicken	0.54	192	25	1.89 (1.00-3.56)	0.05	428	46	1.44 (0.91-2.27)	0.12
Meat	0.94	219	27	1.61 (0.88-2.95)	0.12	422	49	1.65 (1.06-2.56)	0.03

*p* values were adjusted for age, gender, smoking status and BMI. Statistically significant *p* values are indicated in bold type.

Lastly, we looked whether the impact of the *TCF7L2* rs7903146 SNP on anthropometric and glucose-related variables could be modified by dessert and milk intakes in non-T2D subjects (n = 644). No interactions could be observed for milk intake (data not shown). In contrast, there was a nominal interaction between dessert intake and rs7903146 when considering plasma fasting glucose concentrations (*p* = 0.02) (Table 5). The T allele was associated with higher fasting plasma glucose concentrations (*p* = 0.03)only in subjects consuming at least one dessert a day.

**Table 5:** **Impact of the rs7903146 SNP on anthropometric and glucose-related variables in non-T2D subjects, as a function of dessert intake**

		**Non-/low consumers**				**Consumers/high consumers**			
**Parameter**	**Interaction** ***p***	**CC**	**CT**	**TT**	***p***	**CC**	**CT**	**TT**	***p***
N		79	87	41		147	199	88	
Weight (kg)	0.41	72.5 ± 14.0	69.6 ± 15.5	66.5 ± 12.5	**0.04**	71.8 ± 14.0	71.2 ± 15.0	68.3 ± 14.8	0.18
BMI (kg/m^2^)	0.83	26.5 ± 4.9	26.0 ± 5.3	24.7 ± 4.5	0.08	26.3 ± 5.1	25.6 ± 5.0	25.0 ± 5.3	0.06
Waist (cm)	0.60	89.3 ± 12.6	86.0 ± 12.0	84.5 ± 11.8	0.06	87.5 ± 11.9	86.1 ± 12.3	84.2 ± 12.0	0.10
Hip (cm)	0.87	102.0 ± 10.2	101.**5** ± 9.0	99.9 ± 7.9	0.22	102.8 ± 9.4	101.6 ± 9.9	101.0 ± 10.8	0.13
Waist-to-hip ratio	0.24	0.88 ± 0.09	0.85 ± 0.08	0.84 ± 0.08	0.07	0.85 ± 0.08	0.85 ± 0.08	0.83 ± 0.08	0.28
Fasting glucose (mmol/L)	0.02	4.84 ± 0.49	4.81 ± 0.54	4.66 ± 0.53	0.29	4.78 ± 0.51	4.72 ± 0.48	4.89 ± 0.46	**0.03**
Fasting insulin (μIU/mL)	0.24	9.27 ± 7.69	8.83 ± 6.94	6.40 ± 3.83	0.20	8.21 ± 6.05	7.51 ± 4.54	7.10 ± 4.08	0.99
HOMA-IR	0.12	2.0 ± 1.5	1.9 ± 1.7	1.4 ± 0.9	0.16	1.8 ± 1.3	1.6 ± 1.0	1.6 ± 1.0	0.67
HOMA-B	0.41	167.5 ± 189.6	150.9 ± 125.5	120.7 ± 72.9	0.77	157.3 ± 163.1	141.9 ± 106.0	111.6 ± 72.8	0.08

*p* values were adjusted for age, gender, smoking status and physical activity (plus BMI for glucose and insulin-related variables). Statistically significant *p* values are indicated in bold type.

## Discussion

Genetic variants in *TCF7L2* are strongly associated with the T2D risk in many populations [7]. In the present study, we replicated the association between the *TCF7L2* rs7903146 polymorphism and the T2D risk in a population sample from the city of Oran (Algeria).

In the literature, the minor allele frequency of the rs7903146 SNP ranges from 0.30 in Europeans to 0.032 in Asians [20] and 0.42 in Tunisians [10]. The latter value is very close to the frequency observed here (0.43) for an Algerian population.

Genome-wide association studies in populations of European descent have shown that *TCF7L2* is the T2D-predisposing gene with the largest effect reported to date [5,7]. A meta-analysis of 35 studies confirmed the association between the rs7903146 SNP and T2D in a variety of populations, with an overall, homogeneous OR (95% CI) of 1.97 (1.79-2.16) [20]. Although Algerian populations are generally more genetically diverse than European, Asian or indigenous American populations, our results extend the list of populations in which the rs7903146 SNP is associated with T2D risk. Indeed, Northern Africa’s strategic location at the crossroads between Europe, the Middle East and the rest of Africa means that Northern Africans display various combinations of five distinct ancestries. Henn *et al*. have observed an East-to-West increase in the likelihood of autochthonous Maghrebi ancestry across Northern Africa [21]. In the Algerian population studied here, the rs7903146 polymorphism was associated with a 62% increase in the T2D risk. Therefore, *TCF7L2* is a population-independent susceptibility locus for T2D in Europeans [5], African-Americans [22], Arabs (for a review, see [23]) and Algerians (the present work).

To better understand the impact of the *TCF7L2* rs7903146 SNP on T2D, we also searched for associations with quantitative metabolic risk factors. The literature data show that the rs7903146 SNP is associated with fasting glucose concentrations, fasting insulin concentrations and the HOMA-B index [24,25]. In the ISOR population, T allele carriers tended to have lower HOMA-B than C allele carriers (*p* = 0.08) and thus lower insulin secretion, which may explain the association with T2D.

In the present study, the T risk allele was also associated with a lower body weight, BMI, abdominal circumference and waist-to-hip ratio. This observation has been made in a number of population samples [4,24,26,27] but contrasts with another study in which the risk allele was associated with higher BMI [28]. Interestingly, *TCF7L2* is expressed in adipose tissue and is involved in Wnt-dependent regulation of adipogenesis [29]. Moreover, an investigation of the association of *TCF7L2* variants with body fat composition and ectopic lipid storage after a nine-month lifestyle intervention (weight loss) showed that the *TCF7L2* rs7903146 SNP had a negative impact on changes in BMI, non-visceral fat and visceral fat [30].

We also explored the possible influence of diet on T2D risk, as dietary factors have a key role in the development of the condition. Indeed, European and Northern African diets differ significantly in terms of their qualitative composition [31]. The most striking differences usually concern the proportion of fat in the energy intake. European populations have a high fat intake, whereas Northern African populations have a high carbohydrate intake [31]. The Diabetes Prevention Program [26] and the Finnish Diabetes Prevention Study [32] provided the first indications that environmental or lifestyle factors might influence the genetic effect of *TCF7L2* polymorphisms. Moreover, other studies have reported interactions between *TCF7L2* variants and dietary wholegrain products [33], the glycemic index, glycemic load [34] and polyunsaturated fatty acids [35] when considering the risks of T2D and atherogenic dyslipidemia. In the present study, we observed that the T risk allele of the rs7903146 SNP was associated with an even greater increase in T2D risk (OR = 2.61) in subjects with a high dessert intake (i.e. mainly sugary foods). This association might be related to the higher fasting plasma glucose concentrations observed in these subjects. Our present results are in line with Grau *et al*.'s report that the *TCF7L2* rs7903146 SNP makes individuals differentially sensitive to the carbohydrate and fat components of the diet [36]. Investigation of the mechanisms by which *TCF7L2* rs7903146-macronutrient interactions affect the T2D risk might yield new insights into the molecular basis of diabetes and thus provide opportunities for more targeted preventive and therapeutic interventions.

Our study presented some limitations. Firstly, there were few subjects with T2D (n = 76) in the ISOR study, which lowers the study’s statistical power and reduces the ability to detect significant associations with the T2D risk. Using a threshold of p ≤ 0.05, the ISOR study had a statistical power of at least 80% to detect odds ratios ranging between 1.60 and 1.95 for allele frequencies ranging from 0.40 to 0.10 respectively. The effect size of rs7903146 was large enough to enable us to detect a significant association with T2D. In contrast, the number of subjects without T2D (n = 644) was too low to enable us to detect significant associations with fasting glycemic traits (except when diet was taken into account). Secondly, dietary consumption was assessed using a weekly food frequency questionnaire; portion sizes were not taken into account, which prevented us from calculating the quantities of food types consumed. We acknowledge that our data on gene-diet interactions are preliminary and need to be replicated. However, we did not have access to other populations with a similar ethnic background, and were thus unable to replicate our findings. Lastly, the cross-sectional nature of the ISOR study enables associations to be identified but cannot provide information on causality.

## Conclusions

Even though Algerian populations have a heterogeneous genetic background, our present results showed that the *TCF7L2* rs7903146 polymorphism modulates the T2D risk. Furthermore, we found that a high dessert intake increased the T2D risk even more in risk allele carriers. Given that the diet in urban areas of Algeria is evolving towards the greater consumption of sugary foods, it will be important to take our findings into account (if replicated in independent cohorts) in attempts to combat the rising prevalence of T2D.

## Abbreviations

ADA: American Diabetes Association
DBP: Diastolic blood pressure
GLM: General linear model
GLP-1: Glucagon-like peptide 1
SBP: Systolic blood pressure
SNP: Single nucleotide polymorphism
TCF7L2: Transcription factor 7-like 2
T2D: Type 2 diabetes
